# Impacto da Primeira Onda da Pandemia de COVID-19 na Cirurgia Cardiovascular no Brasil: Análise de um Centro Terciário de Referência

**DOI:** 10.36660/abc.20210235

**Published:** 2022-03-10

**Authors:** Luiz Augusto Lisboa, Omar Asdrúbal Vilca Mejia, Elisandra Trevisan Arita, Gustavo Pampolha Guerreiro, Lucas Molinari Veloso da Silveira, Carlos Manuel de Almeida Brandão, Ricardo Ribeiro Dias, Luís Roberto Palma Dallan, Leonardo Miana, Luiz F. Caneo, Marcelo Biscegli Jatene, Luís Alberto Oliveira Dallan, Fabio Biscegli Jatene

**Affiliations:** 1 Instituto do Coração Faculdade de Medicina Universidade de São Paulo São Paulo SP Brasil Instituto do Coração Faculdade de Medicina da Universidade de São Paulo – Divisão de Cirurgia Cardiovascular, São Paulo, SP – Brasil

**Keywords:** COVID-19, Cirurgia Torácica, Hospitalização, Atenção Terciária à Saúde, Mortalidade, Procedimentos Cirúrgicos Eletivos, Cardiopatias Congênitas/cirurgia, Pandemia

## Introdução

A nova infecção respiratória viral causada pela doença de coronavírus 2019 (COVID-19), inicialmente conhecida como 2019-nCoV, surgiu no final de dezembro de 2019 em Wuhan, China, e rapidamente se espalhou pela Ásia, Europa e EUA, caracterizando uma situação de pandemia.^1^ Em maio de 2020, a Organização Mundial da Saúde declarou o Brasil um novo epicentro da pandemia de coronavírus.

Com os níveis alarmantes de disseminação e gravidade da COVID-19, grandes interrupções nos serviços hospitalares de rotina ocorreram à medida que os hospitais se ajustavam com a finalidade de aumentar a capacidade de atendimento aos pacientes com SARS-CoV-2.^2^ Nesse contexto, foram adiadas as cirurgias eletivas com a finalidade de otimizar recursos de saúde e questões de pessoal e de proteger os pacientes da transmissão viral hospitalar.^3^

Embora tenham sido observadas redução do volume cirúrgico e maiores taxas de mortalidade em pacientes operados durante a primeira onda do período pandêmico, ainda não tem sido completamente documentado e compreendido o impacto causado pela pandemia de COVID-19, especificamente na cirurgia cardiovascular.^4^ Com o objetivo de esclarecer essas questões e embasar ações futuras para a retomada das unidades de cirurgia cardiovascular, foi realizada uma análise retrospectiva dos dados cirúrgicos em um centro de referência de grande volume de cirurgia cardiovascular no Brasil, o epicentro da pandemia COVID-19 na América Latina.

## Pacientes e Métodos

No presente estudo retrospectivo, foi utilizado o banco de dados institucional para revisar todos os pacientes submetidos à cirurgia cardiovascular em 2019 e 2020. Foram comparados os desfechos de dois períodos: o período de 1º de março a 31 de julho de 2019 e o outro de 1º de março a 31 de julho de 2020, este segundo incluindo o estágio inicial e o pico da primeira onda da pandemia COVID-19 no Brasil. O presente estudo foi aprovado pelo conselho institucional sob número 4.487.975 em 4 de janeiro de 2021.

Os pacientes incluídos neste estudo foram submetidos à cirurgia cardiovascular maior para adultos ou cirurgia cardíaca congênita e foram categorizados em três estados pré-operatórios: cirurgia eletiva, urgente e de emergência, de acordo com a definição de EuroSCORE II.^5^ Foram excluídos da presente análise pacientes submetidos a transplante cardíaco ou procedimentos de resgate.

Os desfechos primários foram o volume geral de cirurgia cardiovascular e a mortalidade hospitalar, comparando os dois períodos selecionados e os diferentes meses do começo de 2020. A mortalidade hospitalar incluiu pacientes que foram a óbito num período de 30 dias após operação e aqueles que foram a óbito mais tarde durante o período de hospitalização.

### Análise estatística

As variáveis categóricas foram expressas como frequências e porcentagens e comparadas pelo teste qui-quadrado de Pearson ou teste exato de Fisher entre os períodos. Foi considerado estatisticamente significativo α bilateral inferior a 0,05. Todas as análises estatísticas foram realizadas com SPSS, versão 25.0.

## Resultados

**Figura 1:**
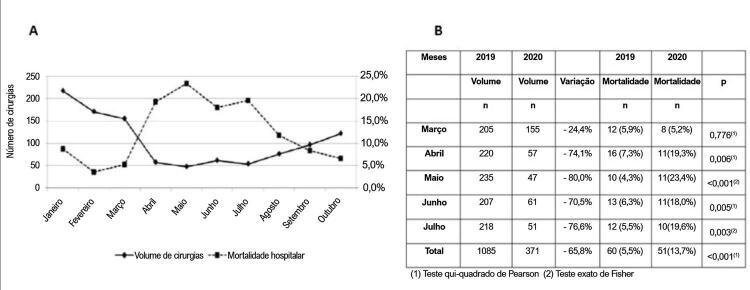
Painel A: Impacto da primeira onda do período pandêmico com redução do volume de cirurgia cardiovascular e aumento da mortalidade hospitalar pós-operatória em 2020. Painel B: Volume de cirurgia cardiovascular e mortalidade hospitalar, comparando março a julho de 2019 e 2020 (período pandêmico).

De 1º de janeiro a 31 de outubro de 2020, 1.056 pacientes foram submetidos à cirurgia cardiovascular em nosso instituto. Com o avanço da pandemia no início de 2020, houve redução no número de cirurgias a partir de março, com retomada a partir de julho. Em janeiro de 2020, foram realizadas 218 cirurgias cardiovasculares e, no auge da pandemia, em maio, apenas 47 cirurgias cardiovasculares foram realizadas. Em outubro, o volume cirúrgico voltou a 122 procedimentos cardiovasculares. A mortalidade hospitalar pós-operatória global também mudou significativamente, de 7,8% em janeiro para 23,4% em maio, e voltou a 6,6% em outubro de 2020 ( Figura 1 , Painel A).

Comparando o período pandêmico (março a julho) de 2020 com o mesmo período (março a julho) de 2019, houve uma redução de 65,8% (de 1.085 para 371, em 2019 e 2020, respectivamente) no número total de cirurgias cardiovasculares realizadas. Em março de 2020 (período inicial da pandemia), a redução no volume de cirurgias cardiovasculares foi de 24,4% e, em maio de 2020 (pico pandêmico), foi de 80,0%. A mortalidade hospitalar pós-operatória teve uma correlação inversa, com um aumento significativo de 5,5% (março a julho de 2019) para 13,7% (março a julho de 2020), p < 0,001. Em maio de 2019, a mortalidade hospitalar pós-operatória foi de 4,3% e, em maio de 2020 (pico pandêmico), foi de 23,4% (p < 0,001) ( Figura 1 , Painel B).

A condição pré-operatória também mudou durante o período pandêmico. Em 2019, cerca de dois terços (66,4%) dos procedimentos de cirurgia cardiovascular eram eletivos. Durante o período pandêmico, houve inversão e cerca de dois terços (65,2%) dos procedimentos cirúrgicos foram de urgência ou de emergência. No pico da pandemia (maio de 2020), os procedimentos de urgência ou emergência representaram 85,1% do total de cirurgias cardiovasculares, com redução de 95,5% nos procedimentos eletivos. No entanto, ambas as condições cirúrgicas diminuíram durante o período pandêmico. Os procedimentos eletivos diminuíram 82,1% e os procedimentos de urgência ou emergência diminuíram 33,7% ( Material Suplementar, Tabela S1 ). Com o aumento da proporção de procedimentos de urgência e de emergência no período pandêmico, em relação ao mesmo período de 2019, também houve aumento do risco cirúrgico (EuroSCORE II) de 2,02 para 7,82 entre os pacientes submetidos à cirurgia de revascularização do miocárdio; de 3,04 para 9,22 entre os pacientes submetidos à cirurgia valvar e de 2,90 a 9,70 entre os pacientes submetidos à cirurgia combinada de revascularização miocárdica e da valva aórtica.

A análise específica das cirurgias cardiovasculares mais realizadas, durante o período pandêmico, confirmou uma redução média de 70% no volume cirúrgico, independentemente do tipo de cirurgia cardíaca. No entanto, o aumento da mortalidade hospitalar foi diferente a depender do tipo de procedimento realizado. Entre as cirurgias por doenças cardíacas adquiridas (cirurgia de revascularização do miocárdio, cirurgia valvar e cirurgia aórtica), a mortalidade hospitalar observada foi significativamente maior. No entanto, em relação às cirurgias cardíacas congênitas, não foi significativo o aumento da mortalidade hospitalar observada ( Material Suplementar, Tabela S2 ).

Levando em consideração apenas o período pandêmico (março a julho de 2020), 39/357 (10,9%) pacientes apresentaram COVID-19 pós-operatória, da maneira seguinte: 13/99 (13,1%) entre os pacientes submetidos à revascularização do miocárdio, 14/79 (17,7%) entre os submetidos à cirurgia valvar, 8/48 (16,7%) entre os submetidos à cirurgia aórtica e 2/113 (1,8%) entre os submetidos a cirurgias cardíacas congênitas. Os pacientes que tiveram COVID-19 tiveram mortalidade hospitalar significativamente maior do que aqueles que não tiveram (35,9% versus 11,6%, p < 0,001). Porém, mesmo aqueles que não tiveram COVID-19 ainda apresentaram mortalidade hospitalar maior, quando comparada ao mesmo período de 2019 (11,6% versus 5,3%, p < 0,001) ( Tabela 1 ).

**Tabela 1:** Mortalidade hospitalar de pacientes com e sem COVID-19, em procedimentos de cirurgia cardiovascular frequentemente realizados, durante o pico do período pandêmico (março a julho) de 2020n (%)

Cirurgia	Total Março a julho 2020		Com COVID-19		Sem COVID-19		Total Março a julho 2019		Com versus sem COVID-19 (2020)	2019 versus sem COVID-19 (2020)

	Volume	Mortalidade	Volume	Mortalidade	Volume	Mortalidade	Volume	Mortalidade	p	p

	n	n (%)	n	n (%)	n	n (%)	n	n (%)		
CRM	99	13 (13,1%)	13	5 (38,5%)	86	8 (9,3%)	325	9 (2,8%)	0,013^(2)^	0,013^(2)^
Valvar	79	9 (11,4%)	14	3 (21,4%)	65	6 (9,2%)	318	12 (3,8%)	0,194^(2)^	0,098^(2)^
CRM + Valvar	18	6 (33,3%)	2	2 (100%)	16	4 (25,0%)	37	4 (10,8%)	0,098^(2)^	0,224^(2)^
Aórtica	48	13 (27,1%)	8	2 (25,0%)	40	11 (27,5%)	111	12 (10,8%)	1,000^(2)^	0,012^(1)^
Congênita	113	10 (8,8%)	2	2 (100%)	111	8 (7,2%)	271	19 (7,0%)	0,007^(2)^	0,946^(1)^
Total	357	51 (14,3%)	39	14 (35,9%)	318	37 (11,6%)	1062	56 (5,3%)	<0,001^(1)^	<0,001^(1)^

*CRM: cirurgia de revascularização do miocárdio. (1) Teste qui-quadrado de Pearson (2) Teste exato de Fisher*

## Discussão

A análise retrospectiva de um banco de dados nacional representativo de um centro de alto volume no Brasil mostrou redução no volume geral de cirurgias cardiovasculares, com aumento nas taxas de procedimentos de urgência ou emergência, bem como aumento significativo na mortalidade hospitalar pós-operatória durante a primeira onda do período pandêmico de COVID-19.

A prática cirúrgica foi significativamente impactada em todas as especialidades ao redor do mundo durante o período pandêmico. Isso foi devido às medidas que o sistema de saúde, por necessidade, adaptou para atender o aumento da demanda de pacientes com SARS-Cov-2.^3 , 6^

Com a propagação da pandemia e as grandes interrupções nas rotinas hospitalares, houve um aumento na mortalidade observada em procedimentos de cirurgia cardiovascular. COVIDSurg collaborative, uma coorte multicêntrica de cirurgias, incluiu 50 pacientes submetidos à cirurgia cardíaca e a mortalidade em 30 dias foi de 34%, entre os pacientes que tiveram infecção perioperatória por SARS-CoV-2.^4^ No período pandêmico, observamos mortalidade hospitalar de 13,7%, sendo de 35,9% entre os pacientes que tiveram COVID-19 pós-operatória. Embora tenha ocorrido um aumento da proporção de procedimentos de urgência e emergência e aumento do EuroSCORE II durante o período pandêmico, a mortalidade observada ainda foi superior à esperada. Este aumento na mortalidade observada no pós-operatório pode estar associada diretamente à infecção por SARS-CoV-2 e indiretamente devido ao cenário geral de interrupções hospitalares.^4^

Em relação à cirurgia cardíaca congênita, não houve diferenç significativa na mortalidade hospitalar entre os dois períodos (7,0% versus 8,8%). Existem dois motivos que podem justificar esta diferença. Os fluxos hospitalares de cirurgia cardíaca congênita já eram anteriormente separados e permaneceram mais isolados durante a pandemia. Outro fator é que, embora as crianças tenham a mesma probabilidade de serem infectadas pelo SARS-CoV-2, assim como os adultos, elas apresentam menos sintomas e doença menos grave.^7 , 8^

À medida que a pandemia de COVID-19 diminui, muitas instituições passaram a estudar estratégias adequadas para reiniciar a cirurgia cardiovascular de rotina e reavaliar a lista de espera para minimizar a mortalidade durante o período de espera. Todas as recomendações das autoridades de saúde pública em relação à contenção de COVID-19 devem continuar a ser seguidas a fim de minimizar a propagação da doença, garantir a segurança dos pacientes e proteger os profissionais de saúde.^9 , 10^ Os pacientes que estão aguardando cirurgia cardíaca eletiva precisam ser gerenciados de forma proativa, dando prioridade àqueles com anatomia de alto risco ou àqueles cujo estado clínico está se deteriorando.^3 , 4^ Com aprendizado contínuo, troca de informações, dados coletados e resultados conhecidos, podemos implementar uma estrutura de prevenção de incidentes que permite a criação colaborativa de medidas de qualidade e segurança para a próxima etapa de retomada da cirurgia cardiovascular, minimizando problemas durante outra onda de infecção.

## *Material suplementar

Para informação adicional, por favor, clique aqui.


