# Supplementary material for: Impacto da Primeira Onda da Pandemia de COVID-19 na Cirurgia Cardiovascular no Brasil: Análise de um Centro Terciário de Referência
Source: Arq Bras Cardiol. 2022 Mar 10;118(3):663–6. [Article in Portuguese] doi: 10.36660/abc.20210235 (PMC8959023; doi:10.36660/abc.20210235)
Supplement: Supplementary file 1 [file 2021-0235_CC_Tabela-suplementar.pdf]

Table S1. Cardiovascular surgery volume according to preoperative status comparing March – July of 2019 and 2020 (pandemic period).

Months	2019	2020		2019	2020	
	Elective	Elective	Variation	Urgent / Emergency	Urgent / Emergency	Variation
	n	n		n	n	
March	136 (66.3%)	94 (60.6%)	- 30.9%	69 (33.7%)	61 (39.4%)	- 11.6%
April	154 (70.0%)	8 (14.0%)	- 94.8%	66 (30.0%)	49 (86.0%)	- 25.8%
May	155 (66.0%)	7 (14.9%)	- 95.5%	80 (34.0%)	40 (85.1%)	- 50.0%
June	134 (64.7%)	14 (23.0)	- 89.6%	73 (35.7%)	47 (77.0%)	- 35.6%
July	141 (64.7%)	6 (11.8%)	- 95.8%	77 (35.3%)	45 (88.2%)	- 41.6%
Total	720 (66.4%)	129 (34.8%)	- 82.1%	365 (33.6%)	242 (65.2%)	-33.7%

Table S2. Surgery volume of more commonly performed cardiovascular surgery procedures and in-hospital mortality comparing March – July of 2019 and 2020 (pandemic period).

Surgery	2019	2020		2019	2020	
	Volume	Volume	Variation	Mortality	Mortality	p
	n	n		n	n	
**CABG**	325	99	- 76.7%	9 (2.8%)	13 (13.1%)	<0.001(1)
**Valve**	318	79	- 80.1%	12 (3.8%)	9 (11.4%)	0.020(2)
**CABG + Valve**	37	18	- 67.3%	4 (10.8%)	6 (33.3%)	0.063(2)
**Aortic**	111	48	- 69.8%	12 (10.8%)	13 (27.1%)	0.010(1)
**Congenital**	271	113	- 70.6%	19 (7.0%)	10 (8.8%)	0.534(1)

(1) Pearson's chi-square test (2) Fisher's exact test
