# Supplementary material for: Impacto da Primeira Onda da Pandemia de COVID-19 na Cirurgia Cardiovascular no Brasil: Análise de um Centro Terciário de Referência
Source: Arq Bras Cardiol. 2022 Mar 10;118(3):663–6. [Article in Portuguese] doi: 10.36660/abc.20210235 (PMC8959023; doi:10.36660/abc.20210235)
Supplement: Translation

# Translation: Impact of the First Wave of the COVID-19 Pandemic on Cardiovascular Surgery in Brazil: Analysis of a Tertiary Reference Center

**Authors:** Luiz Augusto Lisboa, Omar Asdrúbal Vilca Mejia, Elisandra Trevisan Arita, Gustavo Pampolha Guerreiro, Lucas Molinari Veloso da Silveira, Carlos Manuel de Almeida Brandão, Ricardo Ribeiro Dias, Luís Roberto Palma Dallan, Leonardo Miana, Luiz F. Caneo, Marcelo Biscegli Jatene, Luís Alberto Oliveira Dallan, Fabio Biscegli Jatene

**Affiliations:** 1 Brazil Instituto do Coração Faculdade de Medicina da Universidade de São Paulo – Divisão de Cirurgia Cardiovascular, São Paulo, SP – Brazil

**Keywords:** COVID-19, Thoracic Surgery, Hospitalization, Tertiary Healthcare/trends, Mortality, Elective Surgical Procedures, Heart Defects Congenital /surgery, Pandemics

## Introduction

The novel viral respiratory infection caused by coronavirus disease 2019 (COVID-19), initially known as 2019-nCoV, emerged in late December 2019 in Wuhan, China and quickly spread throughout Asia, Europe, and USA, characterizing the situation as a pandemic.^1^ In May 2020, the World Health Organization declared Brazil a new epicenter of the coronavirus pandemic.

With the alarming levels of spread and severity of COVID-19, major disruptions in routine hospital services have occurred, as hospitals adjust in order to increase the capacity to care for patients with SARS-CoV-2.^2^ In this context, elective surgeries were postponed in order to optimize health resources and staffing issues and to protect patients from in-hospital viral transmission.^3^

Although surgical volume reduction and higher mortality rates have been observed in patients operated on during the first wave of the pandemic period, the impact caused by the COVID-19 pandemic, specifically in cardiovascular surgery, has not yet been fully documented and understood.^4^ Aiming to clarify these questions and to create grounding for future actions of resuming cardiovascular surgery units, a retrospective analysis of surgical data was conducted in a high-volume referral center for cardiovascular surgery in Brazil, the epicenter of the COVID-19 pandemic in Latin America.

## Patients and Methods

In this retrospective study, the institutional database was used to review all patients who had undergone cardiovascular surgery in 2019 and 2020. The outcomes of two periods were compared, one period from 1 March to 31 July 2019 and the other from 1 March to 31 July 2020, which includes the early stage and the first wave peak of the COVID-19 pandemic in Brazil. This study was approved by the institutional review board under number 4.487.975 on 4 January 2021.

Patients included in this study underwent major adult cardiovascular surgery or congenital heart surgery, and they were categorized into three preoperative states: elective, urgent, and emergency surgery, according to the EuroSCORE II definition.5 Patients undergoing heart transplantation or salvage procedures were excluded from this analysis.

The primary endpoints were overall cardiovascular surgery volume and in-hospital mortality, comparing the two selected periods and the different months of early 2020. In-hospital mortality included patients who died within 30 days of operation and those who died later during the same hospitalization period.

### Statistical analysis

Categorical variables were expressed as frequencies and percentages and compared by Pearson’s chi-square test or Fisher’s exact test between periods. A two-sided α of less than 0.05 was considered statistically significant. All statistical analyses were performed with SPSS, version 25.0.

## Results

From 1 January to 31 October 2020, 1,056 patients underwent cardiovascular surgery at our institute. With the advance of the pandemic in early 2020, there was a reduction in the number of surgeries starting in March, with recovery starting in July. In January 2020, 218 cardiovascular surgeries were performed, and at the peak of the pandemic, in May, only 47 cardiovascular surgeries were performed. In October, the surgical volume returned to 122 cardiovascular procedures. The overall postoperative in-hospital mortality also changed significantly, from 7.8% in January to 23.4% in May, and it returned to 6.6%, in October 2020 ( Figure 1 , Panel A).

**Figure 1:** Panel A: Impact of the first wave of pandemic period with reduced cardiovascular surgery volume and increased postoperative in-hospital mortality in 2020. Panel B: Cardiovascular surgery volume and in-hospital mortality, comparing March to July of 2019 and 2020 (pandemic period).

Comparing the pandemic period (March to July) of 2020 with the same period (March to July) of 2019, there was a 65.8% reduction (from 1,085 to 371, in 2019 and 2020, respectively) in the total number of cardiovascular surgeries performed. In March 2020 (early pandemic period), the reduction in cardiovascular surgery volume was 24.4%, and, in May 2020 (pandemic peak), it was 80.0%. Postoperative in-hospital mortality had an inverse correlation with a significant increase from 5.5% (March to July 2019) to 13.7% (March to July 2020), p < 0.001. In May 2019, postoperative in-hospital mortality was 4.3%, and in May 2020 (pandemic peak), it was 23.4% (p < 0.001) ( Figure 1 , Panel B).

Preoperative status also changed during the pandemic period. In 2019, about two thirds (66.4%) of cardiovascular surgery procedures were elective. During the pandemic period, there was an inversion, with about two thirds (65.2%) of surgical procedures being urgent or emergency. In the pandemic peak (May 2020), the urgent/emergency procedures represented 85.1% of total cardiovascular surgeries, with a 95.5% reduction in elective procedures. However, both surgical statuses decreased during the pandemic period. Elective procedures decreased 82.1%, and urgent/emergency procedures decreased 33.7% ( Supplemental Material, Table S1 ). With the increase in the proportion of urgent and emergency procedures in the pandemic period, compared to the same period in 2019, there was also an increase in surgical risk (EuroSCORE II) from 2.02 to 7.82 among patients undergoing coronary artery bypass graft, from 3.04 to 9.22 among patients undergoing valve surgery, and from 2.90 to 9.70 among patients undergoing combined coronary and valve surgery.

Specific analysis of the most commonly performed cardiovascular surgeries, during the pandemic period, confirmed an average reduction of 70% in the surgical volume, regardless of the type of heart procedures. However, the increase in in-hospital mortality was different depending on the type of procedure performed. Among surgeries for acquired heart diseases (coronary artery bypass graft, valve surgery, and aortic surgery), observed in-hospital mortality was significantly higher. However, in congenital heart surgeries, the increase in observed in-hospital mortality was not significant ( Supplemental Material, Table S2 ).

Considering only the pandemic period (March to July 2020), 39/357 (10.9%) patients had postoperative COVID-19, distributed as follows: 13/99 (13.1%) among patients undergoing coronary artery bypass graft, 14/79 (17.7%) among those undergoing valve surgery, 8/48 (16.7%) among those undergoing aortic surgery, and 2/113 (1.8%) among those undergoing congenital heart surgeries. The patients who had COVID-19 had significantly higher in-hospital mortality than those who did not (35.9% versus 11.6%, p < 0.001). However, even those who did not have COVID-19 still had higher in-hospital mortality when compared to the same period in 2019 (11.6% versus 5.3%, p < 0.001) ( Table 1 ).

**Table 1:** In-hospital mortality of patients with and without COVID-19, in commonly performed cardiovascular surgery procedures, during the pandemic period peak (March to July) of 2020.

Surgery	Total March to July 2020		With COVID-19		Without COVID-19		Total March to July 2019		With versus without COVID-19 (2020)	2019 versus without COVID-19 (2020)

	Volume	Mortality	Volume	Mortality	Volume	Mortality	Volume	Mortality	p	p

	n	n (%)	n	n (%)	n	n (%)	n	n (%)		
CRM	99	13 (13,1%)	13	5 (38,5%)	86	8 (9,3%)	325	9 (2,8%)	0,013^(2)^	0,013^(2)^
Valve	79	9 (11,4%)	14	3 (21,4%)	65	6 (9,2%)	318	12 (3,8%)	0,194^(2)^	0,098^(2)^
CRM + Valve	18	6 (33,3%)	2	2 (100%)	16	4 (25,0%)	37	4 (10,8%)	0,098^(2)^	0,224^(2)^
Aortic	48	13 (27,1%)	8	2 (25,0%)	40	11 (27,5%)	111	12 (10,8%)	1,000^(2)^	0,012^(1)^
Congenital	113	10 (8,8%)	2	2 (100%)	111	8 (7,2%)	271	19 (7,0%)	0,007^(2)^	0,946^(1)^
Total	357	51 (14,3%)	39	14 (35,9%)	318	37 (11,6%)	1062	56 (5,3%)	<0,001^(1)^	<0,001^(1)^

*CABG: coronary artery bypass graft. (1) Pearson’s chi-square test (2) Fisher’s exact test*

## Discussion

The retrospective analysis of a national representative database of a high-volume center in Brazil showed a reduction in the overall volume of cardiovascular surgery, with an increase in the rates of urgent or emergency procedures, as well as a significant increase in postoperative in-hospital mortality during the first wave of the COVID-19 pandemic period.

Surgical practice was significantly impacted in all specialties worldwide during the pandemic period. This was due to the adaptations the healthcare system necessarily underwent in order to attend increased demand of patients with SARS-Cov-2.^3 , 6^

With the pandemic spread and the major disruptions in hospital routines, there was an increase in observed mortality in cardiovascular surgery procedures. COVIDSurg collaborative, a multi-center cohort of surgeries, included 50 patients who underwent cardiac surgery, and 30-day mortality was 34%, among the patients who had perioperative SARS-CoV-2 infection.4 In the pandemic period, we observed in-hospital mortality of 13.7%, and it was 35.9% among patients who had postoperative COVID-19. Although during the pandemic period there was an increase in the proportion of urgent and emergency procedures and an increase in the EuroSCORE II, the observed mortality was still higher than the expected mortality. This increase in postoperative observed mortality could be associated directly with SARS-CoV-2 infection and indirectly due to the overall scenario of hospital disruptions.^4^

In congenital heart surgery, there was no significant difference in-hospital mortality between the two periods (7.0% versus 8.8%). Two reasons may explain this difference. In-hospital flows from congenital heart surgery were already separated, and they remained more isolated during the pandemic. Another factor is that, although children are just as likely as adults to become infected with SARS-CoV-2, they have fewer symptoms and less severe disease.^7 , 8^

As the pandemic COVID-19 decreases, many institutions are studying appropriate strategies to restart the routine cardiovascular surgery and reevaluate the waitlist to minimize mortality during the waiting period. All recommendations from public health authorities regarding COVID-19 containment must continue to be followed in order to minimize disease spread, ensure patient safety, and protect health care workers.^9 , 10^ Patients awaiting elective cardiac surgery need to be proactively managed, reprioritizing those with high-risk anatomy or whose clinical status is deteriorating.^3 , 4^ With continuous learning, information exchange, collected data, and known results, we can implement an incident prevention structure that allows the collaborative creation of quality and safety measures for the next step of resuming cardiovascular surgery, minimizing problems during another wave of infection.

## * Supplemental Materials

For additional information, please, click here.
